# Clinical Puzzles and Decision-Making in Antisynthetase Syndrome

**DOI:** 10.7759/cureus.15931

**Published:** 2021-06-25

**Authors:** Yelyzaveta Yehudina, Svitlana Trypilka, Anna Isayeva

**Affiliations:** 1 Department of Rheumatology, Institute of Rheumatology, Kyiv, UKR; 2 Rheumatologist Policlinic Department, Rheumatologist Policlinic Department Communal Non-Commercial Enterprise of Kharkov Regional Council "Regional Clinical Hospital", Kharkiv, UKR; 3 Department of Cardiology, Government Institution “L.T.Malaya Therapy National Institute of the National Academy of Medical Sciences of Ukraine”, Kharkiv, UKR

**Keywords:** antisynthetase syndrome, interstitial lung disease, digital ischemia, intravenous immunoglobulin, rituximab, nintedanib, treatment, sars-cov-2

## Abstract

We present a challenging clinical case of an antisynthetase syndrome (ASS) with a four-year follow-up. The disease debuted with skin manifestations and interstitial lung disease (ILD), then the severe Raynaud's phenomenon came to the fore with the development of occlusive vasculopathy and critical digital ischemia. After the relief of vascular lesions, the severity of the condition was determined by ILD. The use of combined pulse therapy with cyclophosphamide and methylprednisolone, treatment with intravenous immunoglobulin made it possible to reduce the activity of ASS: lung lesion and the progression of vasculopathy. However, after the termination of an unplanned pregnancy, the patient again experienced an exacerbation with ILD progression. It was decided to use rituximab, against which the patient's condition was stabilized. Clinical and laboratory remission was achieved, which was maintained for a year and a half. However, the severe acute respiratory syndrome coronavirus 2 (SARS-CoV-2) pandemic triggered a re-exacerbation of the pulmonary domain of the disease, which forced us to use a nintedanib with a positive clinical and instrumental effect.

## Introduction

Antisynthetase syndrome (ASS) is the most severe subtype of idiopathic inflammatory myopathies (IIM), characterized by the development of non-erosive arthritis, myositis, Raynaud’s phenomenon, mechanic’s hands, interstitial lung disease (ILD), resistance to traditional glucocorticoid (GC) therapy, and existence antibodies directed against an aminoacyl-transfer RNA synthetase [1]. The hallmark of ASS is the presence of myositis-specific anti-synthetase antibodies. One of the most studied and widespread antibodies (up to 75% of all cases of anti-synthetase antibodies) is anti-Jo-1, which is directed against histidyl-tRNA-synthetase [2]. Their detection in patients with IIM is considered as a predictor of the ILD development and a poor prognosis [3]. Nearly 70% of patients with ASS with ILD have detectable anti-Jo-1 antibodies and disease activity is strongly related to the titers of anti-Jo-1 antibodies. To date, other anti-synthetase antibodies are also known: anti-PL7, anti-PL12, anti-KS, anti-OJ, anti-EJ, anti-Zo, antibodies to tyrosyl-tRNA synthetase, etc [2].

ASS occurs in approximately 20%-25% of patients with IIM [4]. ASS has certain phenotypic features that distinguish it from the IIM group as a whole. If the classical debut of IIM is characterized by a progressive increase in muscle weakness or skin syndrome, then with ASS, fever, arthritis/arthralgia, or increasing shortness of breath with mild symptoms of myositis may be observed at the onset. Respiratory symptoms (dyspnea and cough) are present in 40%-60% of patients with ASS [5]. Most reports indicate that the frequency of ILD in ASS is in the range of 70%-95% [1]. Occlusive vasculopathy as an outcome of Raynaud's phenomenon is a rare but serious manifestation of ASS and may herald the onset of severe ILD.

The diagnostic criteria proposed by Connors et al. and Solomon et al. allow the recognition of ASS as a separate entity, which can be useful for both clinical and research purposes [6,7]. Both criteria sets require the presence of an anti-aminoacyl tRNA synthetase and one or more of the above-listed clinical features.

Interest in ASS as part of IIM is driven by the peculiarities of its clinical course, significant manifestation heterogeneity depending on the spectrum of detected antibodies, and the difficulty of diagnosis and further therapy. Earlier, we cited our own observation of the ASS diagnosis with rare clinical manifestations - a severe course of Raynaud's phenomenon with the development of occlusive vasculopathy and acute ischemia of the fingers at the onset of the disease [8,9]. Over the next two years, we continued to supervise this patient and observed the entire diverse spectrum of clinical manifestations and the dynamics of the symptom development of this rare syndrome.

## Case presentation

The 33-year-old female presented to a rheumatologist in May 2017 with complaints of ulceration and pain in digits, chilliness and numbness of the fingers, discoloration of the hand skin, arthralgia, morning stiffness up to one hour, fever up to 37-37.3°C, weakness in the limb muscles, increased fatigue and insomnia. Detailed description of the disease onset in Trypilka and Golovach [8].

She noted these complaints since January 2017. Disease debuted with the symmetrical eyes swelling, erythematous rashes on the body, myalgia, arthralgia and shortness of breath. 

**Figure 1:**
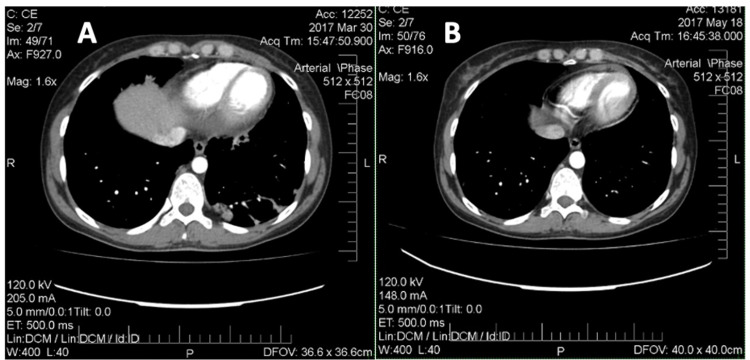
Multislice computed tomography of the chest (2017). A: Signs of bilateral focal pneumonia, multiple infiltrates (March 2017). B: The reverse development of foci in the lungs (May 2017).

Immunological examination revealed positive antinuclear antibodies, anti-RNP and anti-Jo-1. Mixed connective tissue disease was suspected. Methylprednisolone (MPZ) 32 mg/day, methotrexate 10 mg/week, folic acid 5 mg/week and nifedipine 20 mg/day was prescribed. Bilateral pneumonia, infiltrates in the lungs were revealed on multislice computed tomography (MSCT) of the chest (the initial examination of the chest 31/03/2017 (Figure 1A) - signs of bilateral focal pneumonia, multiple infiltrates; re-examining from 18/05/2017 - the reverse development of foci in the lungs was determined (Figure 1B)).

Pain in the joint and muscles reduced however digit cyanosis and extremity coldness increased. Intravenous (IV) infusions of vasoprostane (20 mcg per day №23) were prescribed with no clinical effect. The symptoms of Raynaud's phenomenon prevailed, digital ulceration increased up to the formation of dry necrosis of the fingers. 

During the observation period, attention was drawn to significant changes in laboratory parameters of myositis-specific enzymes: high fluctuations in creatine kinase (CK) values - from 1613 U/L to 854 U/L (normal range: 26-192 U/L).

In May 2017 the diagnosis was revised: the presence of heliotropic edema at the onset of the disease, muscle weakness, episodes of erythematous rashes on the trunk, severe Raynaud's phenomenon, occlusive vasculopathy (digital ulcers, critical limb ischemia, dry necrosis of the distal phalanges of the hands), ILD, laboratory changes (increased CK in a series of tests, positive IgG antibodies to Jo-1), the patient was suspected of dermatomyositis and it’s a special phenotypic variant - ASS. 

The patient was prescribed combined pulse therapy with cyclophosphamide (CYC) (1000 mg) and MPZ (1000 mg for three consecutive days), than prednisone 60 mg per day; iloprost, doxazosin 2 mg two times, sildenafil 25 mg three times a day, rosuvastatin 10 mg once a day, clopidogrel 75 mg per day, alendronate 75 mg per week, calcium and vitamin D. 

**Figure 2:**
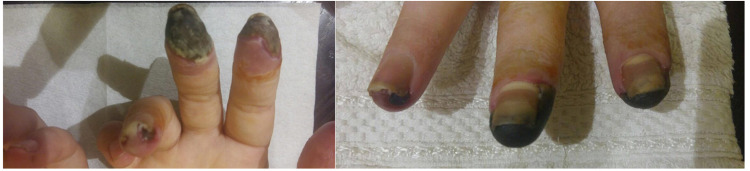
Hands of the patient with antisynthetase syndrome, acute digital ischemia of the fingers, with the development of dry necrosis of the digits (July 2017).

Disappointingly, this therapy did not bring the desired results: the patient developed dry necrosis of the distal parts of the fingers hands (Figure 2), which progressed over time.

**Figure 3:**
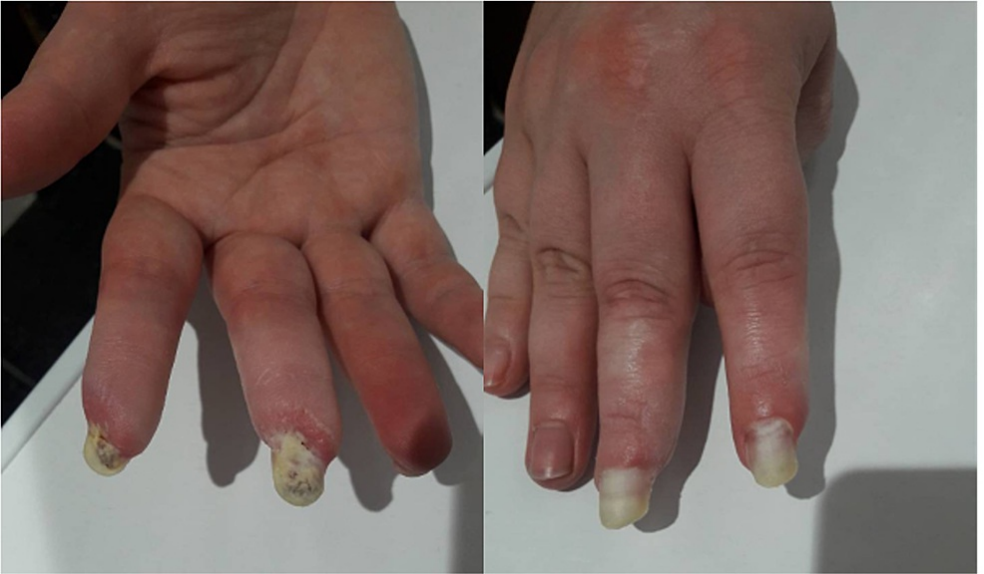
Hands of the patient with antisynthetase syndrome: healing of ulcers, reduction of digital ischemia, replacement of the necrotic zone with areas of regenerated skin (May 2018).

Considering progressive vascular disorders, digital ischemia, formation of digit necrosis, it was decided to start therapy with intravenous immunoglobulin (IVIG) in a total dose of 90 g for three consecutive days one time per month. Against the background of this therapy, there was a gradual positive dynamics in relation to vascular disorders (Figure 3).

**Figure 4:**
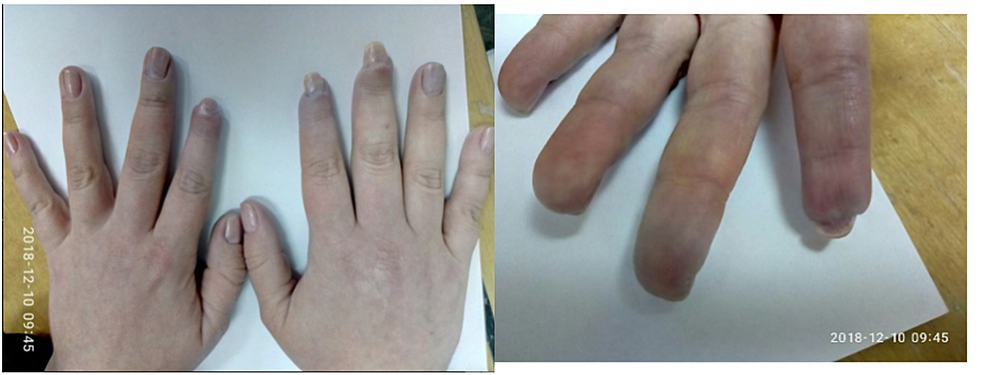
Positive dynamics changes of occlusive vasculopathy in the digits (December 2018).

After eight months of therapy with IVIG, we achieved stable positive dynamics, which allowed us to gradually minimize the dose of oral glucocorticoids (GCs) to 4 mg of MPZ per day; in July 2018 - a decrease IVIG to a minimum maintenance dose of 10 grams per month. In the objective status, there were no pathological abnormalities in the joints, lungs, cardiovascular system, gastrointestinal tract, there was a clear positive dynamic with restore damage hands areas by healthy tissue (Figure 4). Myositis-specific enzymes were steadily kept within the laboratory norm, clinical and laboratory activity was not observed. The condition was regarded as remission.

However, in November 2018, during the follow-up consultation, the patient made the doctor knows about her pregnancy four to five weeks of gestation. The multidisciplinary team meeting decided to terminate pregnancy for medical reasons, taking into account the recent intake of cytostatic drugs (cyclophosphamide), significant vascular disorders.

**Figure 5:**
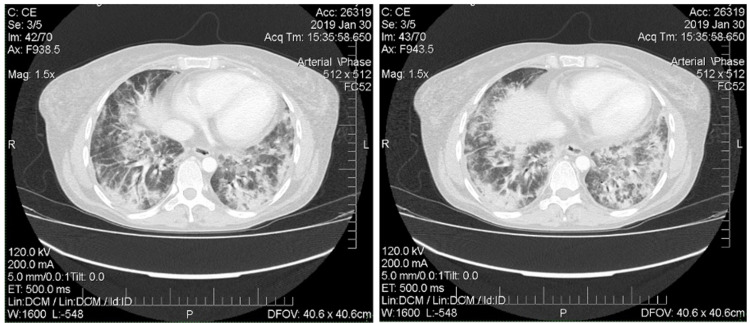
Multislice computed tomography of the chest (January 2019). Horizontal tomography slices on different levels of thorax demonstrate confluent shadows and foci of lung tissue infiltrate from 3 to 45 mm in diameter without clear contours.

In January 2019, the patient's condition worsened significantly: there was an increasing shortness of breath, dry cough, severe weakness, arthralgia, an increase in CK to 567 U/L. There were multiple confluent shadows and foci of lung tissue infiltrate from 3 to 45 mm in diameter without clear contours in the lung parenchyma, mainly in the middle and lower lobes on the right, in the lingual segments and the lower lobe on the left on the MSCT scan of the thorax (Figure 5). The pulmonary pattern is enhanced, enriched. Subsegmental bronchi are indurated.

The patient resumed monthly courses of combined pulse therapy with MPZ 500 mg № 3 and CYC 750 mg monthly, the dose of oral MPZ is increased to 40 mg per day with slight positive dynamics. However, in May 2019, the patient's condition worsened, shortness of breath increased at rest. It was decided to start rituximab (RTX) therapy at a dose of 1000 mg IV twice a month. After RTX administration a positive dynamic was noted: significant decrease in dyspnea, an improvement in general well-being.

**Figure 6:**
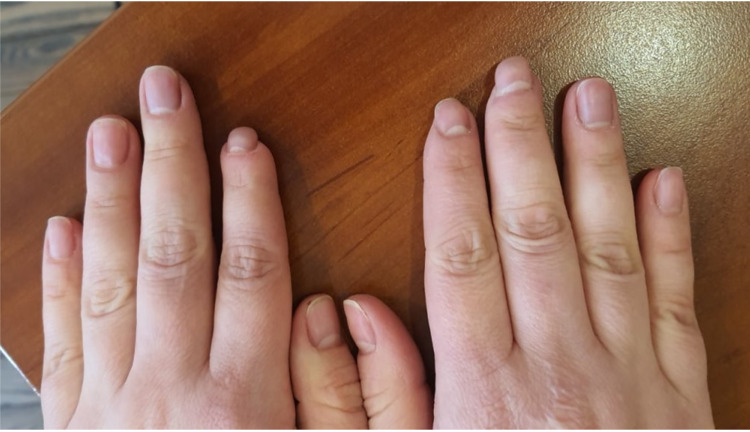
Positive dynamics changes of occlusive vasculopathy in the digits (July 2020).

**Figure 7:**
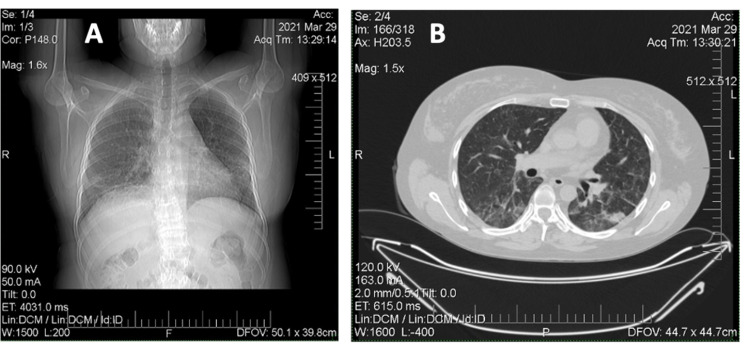
A, B: Multislice computed tomography of the chest (March 2021). A: Vertical tomography slices. B: Horizontal tomography slices. Pronounced thickening of the peribronchial interstitial lung parenchyma with coarse fibrous layers, predominantly located in the hilar regions and lower lobes of the lungs. The lumen of the subsegmental bronchi of the lower lobes is moderately widened - traction bronchiectasis is formed. Negative dynamics in the form of developing pulmonary fibrosis, the formation of traction bronchiectasis.

**Figure 8:**
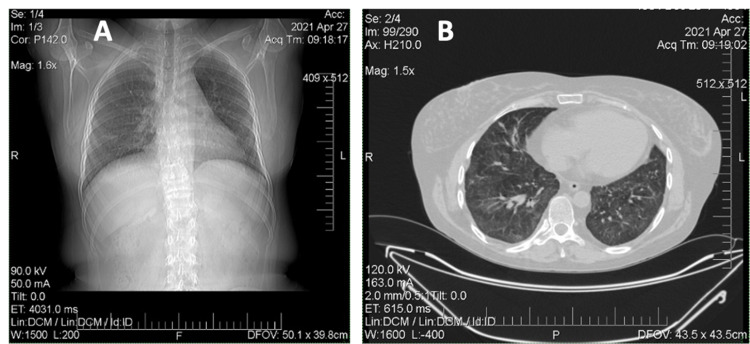
A, B: Multislice computed tomography of the chest (April 2021). A: Vertical tomography slices. B: Horizontal tomography slices.  In the parenchyma of both lungs, mainly in the hilar and posterior regions, there is a thickening of the interlobular interstitium with fibrous layers. The lumen of the bronchi is not expanded. In the previous study, an expansion of the subsegmental bronchi of the lower lobes was noted.

Later, over a period of 2020, the patient received RTX infusion 1000 mg IV every two weeks twice in January and July 2020. Clinical and laboratory remission remained (Figure 6) the dose of MPZ was reduced to 4 mg per day, therapy with bisphosphonates, calcium and vitamin D, doxazosin and sildenafil continued. In December 2020, the patient becomes ill with SARS-CoV-2 infection, with confirmation by PCR and ELISA methods. As a result of which an exacerbation of the disease course developed: worsening of respiratory failure in the form of severe dyspnea and low level of forced volume capacity (FVC) (34%). The dose of MPZ was increased to 40 mg per day. A decision to conduct routine RTX infusions was made in February 2021. However, there is no clinical improvement. MSCT of the lungs revealed negative dynamics in the form of developing pulmonary fibrosis with the formation of traction bronchiectasis (Figure 7). Given the complexity of the situation, we decided to start therapy with nintedanib at a dose of 150 mg two times a day. Already after two weeks of therapy, the patient noted an improvement in the condition in the form of a significant decrease in shortness of breath, which was confirmed by pulmonary function testing (PFT) - FVC 56%. In a control lung MSCT scan after a month of treatment, positive dynamics noted (Figure 8). The patient continues nintedanib therapy.

 

## Discussion

ASS is a rare autoimmune disease characterized mainly by ILD, myositis and arthritis, reported in 90% of cases [10]. However, other manifestations such as fever, rash, and Raynaud's syndrome are less common [10].

Our case was diagnosed in accordance with Solomon et al. (2011) criteria that require the presence of anti-synthetase antibodies with two major criteria - ILD (not attributable to another cause) and polymyositis or dermatomyositis by Bohan and Peter criteria [7].

However, in the clinical case of our patient, there is a very rare manifestation of ASS - occlusive vasculopathy with the development of critical digital ischemia with the rapid formation of fingertip necrosis. 

ASS onset may be “complete” or “incomplete”. The complete form of ASS includes the triad of ILD, myositis, and arthritis, but it is reported only in 19.5% of cases at the onset of the disease [11]. A patient with an incomplete picture of the disease may develop other clinical manifestations over time. Trallero-Araguás et al. studied clinical manifestations and long-term outcomes in 148 patients with anti-Jo-1 syndrome. In most cases, at the onset, ASS had an incomplete picture: isolated ILD - 32.4%, isolated myositis - 26.9%, and isolated arthritis - 17.9% of patients [12]. Only a minority of patients had stable disease course by the end of the follow-up period, isolated ILD was still noted in 14.5% of patients, isolated myositis - in 15.9%, and isolated arthritis - in 2.1% [12]. The median time of occurrence of a new triad manifestation was 14 months (independently from the presence of accompanying findings). It is also reported that patients with ASS have the mortality rate four times higher than in the population [12].

Literature data driving ASS treatment are poor and mostly based on the case series or on small cohorts of patients. There is no standardized regimen for ASS treatment; however, treatment with prednisone 1 mg/kg/day (or MPZ 1 g IV daily for three days, in severe cases) is the most common initial therapy [10]. Additionally, a steroid-sparing agent can be used along with GC and has shown a better survival and fewer relapses in comparison to prednisone monotherapy. CYC is worldwide used in clinical practice (generally IV pulses 0.3-1.5 g/m^2^ or 10-15 mg/kg administered at weekly to monthly intervals for 6-12 months) and the prevalence of ongoing CYC pulses is about the 25% of patients in American, European NEtwork of Antisynthetase Syndrome (AENEAS) cohort [11]. We chose CYC guided by the proposed approach algorithm for severe disease by Oddis and Aggarwal [13].

Our clinical case illustrated the effectiveness of IVIG in severe occlusive vasculopathy, as a result of which it was possible to achieve the reverse development of dry necrosis, restoration of viable tissues and relief of the digital ischemia. IVIG in IIM is beneficial when there is esophageal involvement, refractory rash, pregnancy, active infection, and calcifications but evidence of its use in myositis-associated ILD is limited to case reports only [14]. IVIG can be used alone as a first-line, second-line or third-line therapy or as a concomitant therapy with any drug on the basis of the clinical manifestation or refractoriness of the disease [13].

A decision on a possible pregnancy in such situations should be resolved strictly individually and only after achieving stable clinical, laboratory and instrumental remission. Pregnancy should be planned, and the approach to keeping it balanced and justified after assessing all possible health risks. In our case, it was the hormonal changes associated with pregnancy and its termination triggered the reactivation of an aggressive autoimmune process with lung lesion. During pregnancy many autoimmune diseases go into remission, only to flare again in the early post-partum period. Pregnancy is able to influence the onset and progression of autoimmune and inflammatory diseases by influencing the T cell cytokine-mediated responses during the gestation period, the post-partum period, but also decades after the pregnancy period [15].

So, we used "therapy of despair" applying RTX and obtained a positive result as all possible treatments had not been shown to be effective. According to current guidelines and previous studies, rituximab should always be considered for refractory IIM and refractory ILD. Increasing number of retrospective studies have shown the benefit and safety of RTX in ILD associated with ASS with reported objective improvement in PFT, ground-glass opacities and stability, or fibrosis on high-resolution computer tomography (HRCT) of chest. In an open-label study of 10 patients with ASS and refractory ILD, treatment with RTX resulted in a substantial steroid-sparing effect; clinically significant increase in FVC and/or DLCO was observed in five out of the 10 patients, stabilization in 4, and worsening in 1 [16]. In another retrospective study of 17 anti-Jo-1-positive ASS patients who received RTX, 16 showed a more rapid and marked response than the others who were treated with conventional immunosuppressants (including CYC, cyclosporine, azathioprine, methotrexate, and leflunomide) [17].

However, the new realities and the COVID-19 pandemic made adjustments in the disease course of our patient, and, despite the achieved remission within a year and a half, we received an exacerbation of ASS, specifically the pulmonary domain after SARS-CoV-2 infection in the form of ILD progression. 

It has been shown that patients with preexisting rheumatic diseases can flare during COVID-19 and develop new manifestations [18]. This situation forced us to think and act outside of the box and we began therapy with nintedanib, which, even in a short observation period, showed its effectiveness on the dynamics of pulmonary changes according to HRCT data.

Nintedanib an oral triple kinase inhibitor targeting pro-fibrotic pathways, has been used for the treatment of idiopathic pulmonary fibrosis (IPF). Based on positive results from phase III, placebo-controlled, randomized comparative clinical trial conducted in patients with systemic sclerosis-associated interstitial lung disease (SSc-ILD), nintedanib received approval for the treatment of SSc-ILD [19]. Indication of nintedanib is expanding from IPF to various forms of ILD with progressive fibrosing phenotype. Phase III clinical trial INBUILD was initiated to investigate the efficacy and safety of nintedanib in patients with ILD with progressive fibrosing phenotype, including those with ILD associated with CTD, such as rheumatoid arthritis, SSc, dermatomyositis/ polymyositis, mixed connective tissue disease, Sjogren’s syndrome, and even ASS. The primary results have been published, and showed that nintedanib significantly reduced the annual rate of decline in FVC over 52 weeks compared with placebo [20].

## Conclusions

Thus, this clinical case is of interest for its long-term observation of different domains of ASS, as the most severe subtype of IIM, and is of greatest interest not only for rheumatologists, but also for doctors of related specialties (pulmonologists, allergists, vascular surgeons). Discussion of such rare clinical cases is necessary to find ways to overcome the difficulties of managing such patients, who are often resistant to conventional IIM therapy. And the COVID 19 pandemic forces us to take into account the possible combination and aggravation of an already serious pathology with new realities, which affects the choice of clinical decisions.
